# Polymorphisms of the IL-17A Gene Influence Milk Production Traits and Somatic Cell Score in Chinese Holstein Cows

**DOI:** 10.3390/bioengineering9090448

**Published:** 2022-09-07

**Authors:** Sahar Ghulam Mohyuddin, Yan Liang, Wei Ni, Abdelaziz Adam Idriss Arbab, Huiming Zhang, Mingxun Li, Zhangping Yang, Niel A. Karrow, Yongjiang Mao

**Affiliations:** 1 Key Laboratory for Animal Genetics, Breeding, Reproduction and Molecular Design of Jiangsu Province, College of Animal Science and Technology, Yangzhou University, Yangzhou 225009, China; 2 Joint International Research Laboratory of Agriculture and Agri-Product Safety of Ministry of Education of China, Yangzhou University, Yangzhou 225009, China; 3 Biomedical Research Institute, Darfur University College, Nyala 63313, Sudan; 4 Center for Genetic Improvement of Livestock, Department of Animal Biosciences, University of Guelph, Guelph, ON N1G 2W1, Canada

**Keywords:** milk production traits, Chinese Holstein cows, IL-17A, single nucleotide polymorphism

## Abstract

The cow’s milk production characteristics are a significant economic indicator in the livestock industry. Serum cytokines such as interleukin-17 (IL-17) may be potential indicators for bovine mastitis concerning the milk somatic cell count (SCC) and somatic cell score (SCS). The current study aims to find previously undiscovered single nucleotide polymorphisms in the bovine (IL-17A) gene and further investigates their associations with milk production traits in Chinese Holstein cows. Twenty Chinese Holstein cows were randomly chosen from six farms in Jiangsu Province, China. The DNA was extracted from selected samples of bloods for PCR amplification Sequence analyses were used to find SNPs in the bovine (IL-17A) gene. The discovered five SNPs are g-1578A>G, g-1835G>A, and g-398T>A in the 5′UTR; g3164T>C and g3409G>C in the exon region. The genotyping of Holstein cows (*n* = 992) was performed based on Sequenom Mass ARRAY and SNP data. The connection between SNPs, milk production variables, and the somatic cell score was investigated using the least-squares method. Based on the results, SNP g-398T>A had a significant linkage disequilibrium with g3164T>C. SNPs were found to have significant (*p* < 0.05) correlations with the test-day milk yield. In conclusion, IL-17A affects cow’s milk production traits significantly.

## 1. Introduction

Mastitis is characterized by various organic compounds and physical and bacterial alterations in the milk, as well as pathological changes in the udder tissues of cattle [1]. It is the most frequent disease in dairy animals, resulting in annual losses of USD 2 billion in the United States and USD 35 billion worldwide [2,3,4]. Despite an increase in milk yield, dairy farmers suffer substantial economic losses due to mastitis, even on very well-organized and modern farms. It has been noted that dairy cows’ susceptibility to mastitis increases as the milk production capacity increases [5]. Furthermore, a lower milk production and quality have resulted in substantial economic losses for the dairy industry [6]. Some udder pathogens play a significant role as potential sources of human infections, food-borne diseases, and intoxications [7,8]. The milk SCS, which is intensely linked to clinical mastitis [9], is utilized as an indirect technique for mastitis reduction. In China, the Holstein cow is the most common dairy cow breed. Milk production traits are one of the essential cost-effective features of Holstein cows and a direct indicator of dairy farm management. The cows’ milk production characteristics are influenced by various factors, including genetic, physiological, and environmental impacts. Some critical features directly impact the milk yield and production capacity [10]. There is a strong correlation in various production metrics, such as fat content and milk yield, protein content, milk urea nitrogen, and somatic cell count [11,12].

The physiology of Holstein cows’ milk production was previously studied [13]. Single nucleotide polymorphisms (SNPs) belongs to third-generation genetic markers. SNPs are alterations in the prevalent DNA sequence when one base in a gene is changed; that is, if one nucleotide differs from the normal sequence. For example, the SNP may result in the substitution of the nucleotide thymine (T) with the nucleotide guanine (G) in a specific location in the DNA [14]. SNPs and genome linkage have been used to identify genes involved in milk production traits in cows [15]. The two key factors that contribute to the development of mastitis are genetics and the environment [16]. The optimum indirect indicators for determining the severity of mastitis are the milk SCC and SCS [17]. The mastitis influence can be lowered through genetic testing and the indirect selection of cattle with lower SCS [18,19]. The serum cytokines, such as interleukin-4 (IL-4), IL-6, IL-17, tumor necrosis factor, and interferon, act as an indirect parameter in inflammatory circumstances [20,21], suggesting that serum cytokines, in addition to SCC and SCS, should be included as essential indicators for bovine mastitis. Previous research has shown that mutations in the IL-17F and IL-17A genes lead to inflammatory disorders such as inflammatory bowel disease [21], asthma [22], rheumatoid arthritis [23], ovarian carcinoma [24], and colon and breast cancer [25,26]. The role of IL-17F and IL-17A progression in animals indicates that they can be candidate genes for mastitis tolerance in cattle. The innate and adaptive immune systems are linked by IL-17. The IL-17 family has six members, with IL-17A (which was synthesized approximately two decades ago) [27]. The predicted amino acid sequence alignment revealed that IL-17A is most comparable to other family members and has similar functions in producing inflammation [28]. Higher levels of IL-17A have been associated with certain types of gastric cancer [29]. They have varying effects on the pathophysiology of ulcerative colitis. IL-17 expression influences cellular recruitment patterns at the site of inflammation and serves as a marker. The estimated protein sequence alignment revealed that IL-17A is the most similar to other family members and triggers an immune response marker of a molecule that similarly regulates neutrophil and eosinophil infiltration [30]. According to previous studies, IL-17 F and IL-17A SNPs were strongly related to the serum cytokine (IL-17 and IL-4) in both Holstein and Sanhe cattle. Additionally, the findings provide the first proof that the cytokine IL-4 of bovine mastitis is highly correlated with the IL-17A promoter polymorphism, whose function is still unknown. According to the study’s findings, SNPs in the IL-17F and IL-17A genes had a comparable impact on Chinese Holstein and Sanhe cattle. In studies on mastitis susceptibility in dairy cattle, the SNPs that exhibit a strong correlation with mastitis indicator traits in the Holstein and Sanhe cattle breed may therefore serve as important genetic markers [31]. The current study aims to assess the IL-17A as a potential gene for association analysis with SCS and milk traits in Chinese Holstein cattle and find out the SNPs in IL-17A that contribute to milk production traits and SCS in Holstein cows in southern China.

## 2. Materials and Methods

### 2.1. Animal Housing

The current study collected 12,085 test day records from 992 Chinese Holstein cows from six different farms in Jiangsu Province, China. All dairy farms were raised in an accessible stall, with three instances of feeding and milking per day, and all were fed with total mixed ration (TMR). Dairy cows are divided into different groups on dairy farms. The typical formula for early lactation cows with a 30 kg daily milk yield is shown in Table 1 [32]. Mixed milk samples were taken from the tested cattle, and 40 mL milk samples were taken from each cow. Sampling was conducted at the ratio of 4:3:3 (morning: middle: evening). The collected samples were sent to the testing laboratory for determination on the same day.

### 2.2. Test Sample

Healthy Chinese Holstein cows on dairy farms in Jiangsu province, China, were randomly chosen from a group of 992 cows, and approximately 5 mL of blood was collected from the tail vein and preserved at −80 °C.

### 2.3. Data and Sample Collection

Dairy cow management, including data collecting, was carried out through DC305 software (Valley Ag. software, San Francisco, CA, USA). The data were selected based on the following criteria to provide both consistency and dependability for statistical analyses: TDMY ranged from 5 to 60 kg, FC from 2% to 7%, PC from 2% to 6%, and SCS from 0 to 9. In the end, 9076 test day records were used in this investigation [33].

### 2.4. Primer Design

The Designer software tool (Primer Premier 5, PP5, Premier, Ottawa, Canada) designed primers for SNP identification inside IL-17A based on the sequence provided in GenBank (Gene ID: 282863, NC_037350.1). Table 1 lists the primer sequences.

### 2.5. DNA Extraction and SNP Genotyping

DNA was isolated from blood using a conventional technique and the phenol-chloroform procedure, then dissolved in TE buffer (Tris + EDTA buffer, used as a dissolving reagent to prevent enzymatic destruction of nucleic acids) [34]. The DNA samples were diluted to 100 ngL^−1^ and stored as frozen at −20 °C for subsequent usage after confirming the quality and concentration of DNA. The optimum annealing temperature was used to determine the PCR temperature gradient (Table 2); in a PTC-200 DNA Engine cycler (Bio-Rad, Big Sur, CA, USA), the PCR reaction was carried out. In order to find the SNP site and its location, 20 samples were randomly selected from 992 cow DNA samples. Sequencing polymerase chain reaction was used to identify all SNPs in bovine IL-17A genes (PCR). The amplification effect was verified using agarose gel electrophoresis, and the result was confirmed by sequence analysis by Sangon Company (Shanghai, China).

### 2.6. Amplification, Sequencing, and Genotyping of a Single DNA Sample

Based on the NCBI, six primers were designed and sequenced by the Shanghai Sangon Company (Shanghai, China). The sequences were then analyzed, and the mutation sites and locations were found using three software programs: SeqMan. The amplification effect was verified using agarose gel electrophoresis, and the Shanghai Sangon Company sequenced the results (Shanghai, China). (Invitrogen, Carlsbad, CA, USA), SnapGene Viewer (Invitrogen, Carlsbad, CA, USA), and Vector NTI (Invitrogen, Carlsbad, CA, USA). Following the discovery of the SNP sites, the MassARRAy technology was used for genotyping all 992 samples (including the prior 20 samples) (Sequenom Inc., San Diego, CA, USA). Twenty samples were conducted twice to confirm the SNP analysis results (the tester was unaware that these twenty samples were repeated). SNP genotyping was shown to be 100 percent accurate in the study.

### 2.7. Statistical Analyses

Individual sequencing data were used to detect all SNP sites, and genotypes and alleles were recorded and calculated at each SNP site. Each polymorphism was assessed for Hardy–Weinberg equilibrium using the chi-square test. SHEsis (http://analysis.bio-x.cn/SHEsisMain.htm) accessed on 3 September 2021 was used for standard population genetics data analysis (including gene frequency, genotype frequency, HWE, and linkage-disequilibrium analysis, among other things) [35,36]. The software Beagle 5.1 was used to determine each cow’s specific haplotype (Brian L. Browning, Washington, DC, USA) [37]. The relationships between milk production traits /SCS (the distribution of SCC is skewed in statistical analysis, so it was transformed into the form of SCS, which follows a normal distribution with SCS = log2 (SCC/100) + 3), and genotypes and haplotypes were investigated using the least squares technique and the general linear model (GLM) of SPSS Ver26.0 (IBM, Armonk, New York, NY, USA) [37,38]. The model was as follows:

Yijklmnop = µ + Yeari + Seasonj + Parityk + CSl + DIMm + Fn + Go + eijklmnop(1)

As mentioned above, Yijklmnop is the dependent variable (here, it refers to TDMY, FC, PC, and SCS); µ stands for the overall mean; Yeari is the fixed-effect of the ith year (i = 2016 to 2018); Seasonj is the fixed-effect of the jth test season spring (March–May) summer (June–August), autumn (September–November), and winter (December–January) and February of the following year; Parityk is the fixed effect of the kth parity (cows parity is 1 to 3); CSl is the fixed effect of the lth calving season (here, the division of calving season coincides with the division in test season); DIMm is the fixed effect of the mth DIM class (DIM is days in milk; here, three levels were divided as <100 d, 100 d to 200 d, >200 d); Fn = the fixed effect of the nth farm (*n* = 6, six different farms from Jiangsu Province, China); Go = the fixed effect of the oth genotype or haplotype; eijklmnop = the random residual effect. At *p* < 0.05, differences were judged as statistically significant. For multiple comparisons among different levels of components, Duncan’s approach was used.

## 3. Results

### 3.1. SNP of Bovine IL-17A Gene (Genotype Frequency, Allele Frequency, and Hardy–Weinberg’s Law)

**Figure 1:**
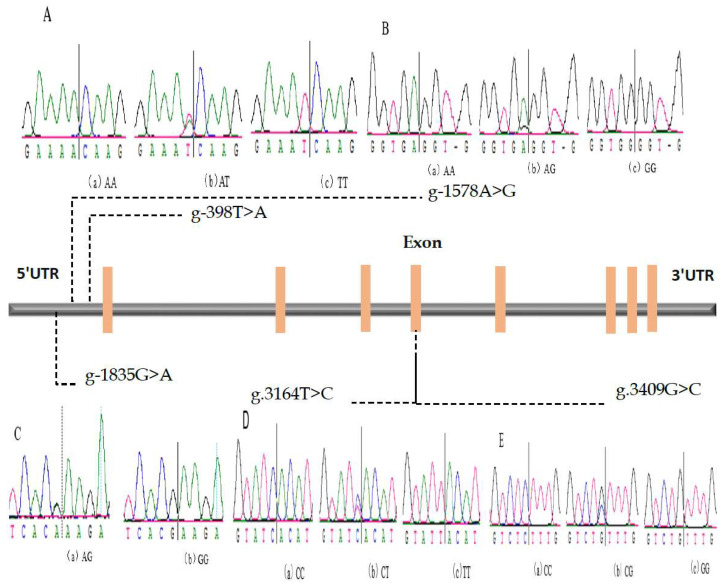
Schematic diagram of the *IL17A* gene with the localization of the five identified SNPs. (**A**): -398T>A; (**B**): -1578A>G; (**C**): -1835G>A; (**D**): 3164T>C; (**E**): 3409G>C.

In the current study, we analyzed the pooled DNA of 20 random Chinese Holstein cows and discovered five novel SNPs in the IL-17A gene (Figure 1). Among them, g-1578A>G, g-1835G>A, and g-398T>A were found in 5′UTR, and g 3164T>C and g 3409G>C were found in the exon region. The genotype and allele frequencies of the five SNPs loci of the IL-17A gene were identified using the Hardy–Weinberg equilibrium and chi-squared test (Table 3). Our results showed that the locus g-398T>A gene frequencies of alleles A and T were 0.514 and 0.486, respectively. The AA, AT, and TT genotype frequencies were 0.247, 0.534, and 0.219, respectively. The gene frequencies of alleles A and at the g-1578A>G locus were 0.374 and 0.626, respectively, representing the G allele as more dominant in the population. The AA, AG, and GG genotype frequency was 0.109, 0.529, and 0.362, respectively. For the g-1835G>A locus, the gene frequencies of alleles A and G were 0.126 and 0.874, making allele G higher and dominant over allele A in the population. The AA, AG, and GG genotype frequency was 0.026, 0.200, and 0.774, respectively. The locus g 3164T>C gene frequencies of alleles C and T were 0.637 and 0.363, respectively, making allele C higher and dominant over allele T in the population. The genotype frequency of CC, CT, and TT was 0.398, 0.479, and 0.123, respectively. The locus g 3409G>C gene frequencies of alleles C and G were 0.488 and 0.512, respectively, making allele G dominant over allele C in the population. The genotype frequency of CC, CG, and GG was 0.383, 0.211, and 0.406, respectively (Table 3). Our results showed that the chi-square test for all five SNPs was in Hardy–Weinberg equilibrium (Table 3). The number of animals with five specific SNPs was 967, 815, 810, 915, and 948 for g. -398T>A, g. -1578A>G, g -1835G>A, g. 3164T>C, and g. 3409G>C, respectively (Table 3).

### 3.2. Haplotype Analysis of Single-SNPS of IL-17A Gene of Chinese Holstein Cows

**Figure 2:**
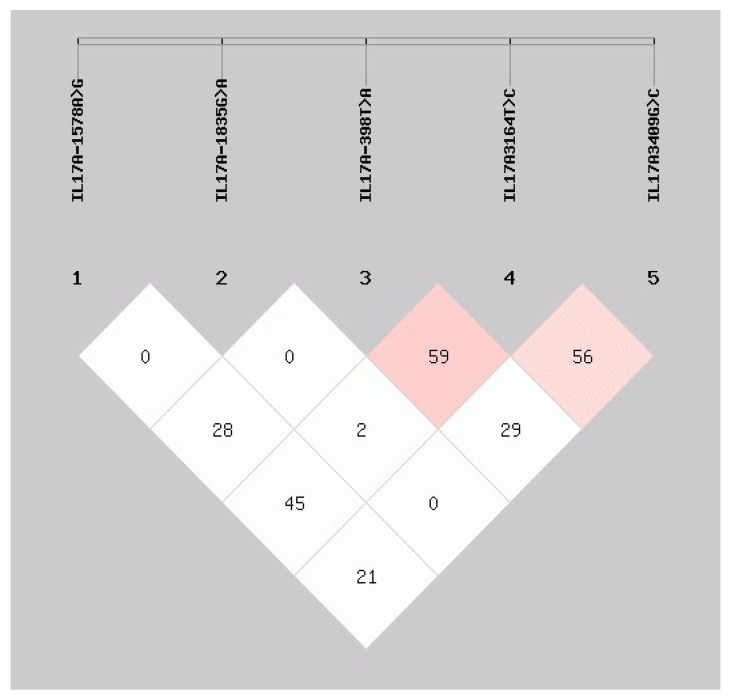
The linkage disequilibrium (LD) among five SNPs of IL17A gene. Note: The values in boxes are pairwise SNP correlations (r2), and the bright red box indicates approximate complete LD (r2 = 1).

The Haploview tool Beagle 5.1 (Brian L. Browning, Washington, DC, USA) was used for the haplotype analysis, and the results revealed that SNPs (g. -398T>A) were connected to milk characteristics. A high linkage was observed between blocks using the linkage disequilibrium analysis (g. -398T>A and g. 3164T>C) for the IL-17A gene (Figure 2); furthermore, 19 haplotypes were reconstructed for the SNPs (Table 4). The highest frequency of haplotype GGACC (0.303) was observed, followed by haplotype AGTTG, with a frequency of (0.281). The r2 value was 0.59 between g. -398T>A and g. 3164T>C, as shown in (Figure 2).

### 3.3. Associations’ of SNPs in IL17A Gene with Milking Traits and Somatic Cell Score

Based on the result, SNPs g. -398T>A and g. 3164T>C were nearly totally linked, so, further, we examined the link of five SNPs (g-1578A>G, g -1835G>A, and g-398T>A were in 5′UTR, and g 3164T>C and g 3409G>C were located in the exon region) with milk traits and SCS. The expected effects of the *IL17A* gene on milk production traits and SCS are shown in Table 5. *IL17A* -398T>A, *IL17A* 3164T>C, and *IL17A* 3409G>C SNP sites had a significant association with only the milk yield, whereas *IL17A*-1578A>G had an association with both the milk yield and SCC. However, there was no association between *IL17A*-1835G>A SNP sites and any measured traits.

The SNP *IL17A*-1578A>G in the 5′UTR had a highly significant effect on the milk yield and SCS (*p* < 0.01). The milk yield of the GA genotype was significantly higher than the AA genotype (*p* < 0.05). Still, there was no significant difference between the GA and GG genotypes, and the AA genotype’s SCS was significantly higher than the GA and GG genotypes (*p* < 0.05). With the increase in A>G, SCS began to trend downward. *IL17A*-398T>A was found to have a statistically significant impact on the milk yield (*p* < 0.05). The TDMY of the AA genotype was significantly higher than those of the TT genotype (*p* < 0.05). The downwards trends in TDMY observed as T>A increased. On TDMY, the SNP *IL17A* 3164T>C had a highly significant effect (*p* < 0.01). The TDMY of the CC genotype was significantly higher (*p* < 0.05) than that of the TC and TT genotypes. With the increase in T>C, TDMY showed a downward trend. CC genotype animals had a significantly higher (*p* < 0.05) milk yield than GG genotype animals in *IL17A* 3409G>C. Similarly, the milk yield in GC cattle was higher than in GG, but there was no significant difference (*p* > 0.05)

## 4. Discussion

Mastitis is the primary infectious disease that affects dairy herds and causes significant economic losses for the milk industry. Subclinical mastitis has been linked to a considerable decrease in milk production, which has resulted in significant financial losses [39]. In addition to its financial impact, mastitis is crucial for maintaining public health.

The IL-17 cytokine family is a relatively new family linked to adaptive and innate immune systems. IL-17A are members of the IL-17 cytokine family, which is essential for the pathogenic activity of IL-17 cells and the production of a variety of proinflammatory mediators in the body [40]. Polymorphisms in the IL-17A and IL-17F cytokines can impact the activity and expression of inflammatory mediators, which can affect interleukin-17 activity (IL) [41,42]. IL-17A polymorphisms have been linked to several malignancies, including gastric and breast cancer. These studies suggested that there is no correlation between the polymorphic locus and production or milk quality parameters. To the best of our knowledge, currently, there is very little evidence available about the significance of interleukin-17A (IL-17A) in milk production. In our investigation, five unique SNPs were discovered in IL-17A in Holsteins, with SNPs g. -398T>A and g. 3164T>C being found in LD. As a result, five of these SNPs were selected for additional screening to determine whether they have any relationships with milk production traits or SCS. According to the current findings, the g. -398T>A and g. 3164T>C SNPs were highly linked with milk production characteristics. To the best of our knowledge, this is the first study to investigate the relationships between SNPs in IL-17A and the milk production traits of Chinese Holstein cows.

The previous studies showed the role of interleukin-17A in the inflammation of cow mammary glands, but the findings have been promising [31]. Somatic cells from a Chinese Holstein cow infected with *Staphylococcus aureus* had transcripts of the interleukin-17A gene [43]. The transcriptome analyses of the early response of bovine mammary epithelial cells, when induced with *S. aureus* culture, revealed that these bacteria elicit the production of IL-17A, a pro-inflammatory cytokine [44]. A further finding was the presence of the bovine receptor for interleukin 17A (IL-17RA), which is related to the proper functioning of this cytokine pathway in healthy mammary epithelial cells [45]. Tassi et al. revealed that cattle with *S. uberis* mastitis produce interleukin-17A (IL-17A) [46]. Findings by Roussel et al. revealed that the IL-17A gene was overexpressed in the udder of cows suffering from *E. coli* mastitis [47]. The authors of this study discovered that interleukin-17A might help mammary epithelial cells to become more effective in fighting off germs after contracting an *E. coli* infection. Furthermore, the results of Usman et al. suggested that IL-17A may be one of the agents responsible for the immune response and mastitis resistance in cattle. It is reported that the IL-17A genes show a substantial correlation with mastitis indicator traits in Holstein cows, suggesting that they could be used as essential genetic markers in a mastitis sensitivity investigation in dairy animals [31]. Furthermore, SCS is a continuous trait that is widely considered as a significant determinant of subclinical mastitis, and various environmental circumstances highly impact it. The present study found that the SNPs in IL-17A significantly correlated with milk production traits and SCS in Holstein cows. The SNPs *IL17A-1578A>G*, *IL17A-398T>A*, *IL17A-398T>A,* and *IL17A 3409G>C* were associated with TDMY. Moreover, we found that the SNPs *IL17A-1578A>G* showed significant effects on SCS. In the previous study, SNPs in IL-17A genes were significantly associated with SCS [31]. The previous studies showed significant changes in SCS based on the age at which the calf was born for the first time. Cows calved sooner (but not before 22 months of age) are likely to have a higher milk yield, and, as previously shown, a higher milk output is connected to an increased SCS [48]. This could be because cows with low AFC levels have compromised immune systems. A previous study discovered that IL-17A, which is known to play an essential role in mucosal immunity against many extracellular and some intracellular pathogens, is also involved in the defense of the mammary gland, which is a non-mucosal tissue lined by a secretory epithelium [49,50]. The findings presented in the above hypothesis support the concept that IL17A plays a significant role in the synthesis of milk fat. According to the findings of the studies mentioned above, SNPs in the *IL17A* gene have a considerable impact on the lactation performance of Holstein cows.

## 5. Conclusions

In conclusion, despite these limitations, the present analysis demonstrates that the *IL17A*-398T>A, *IL17A* 3164T>C, and *IL17A* 3409G>C SNP sites had a significant association with only the milk yield, whereas *IL17A*-1578A>G had an association with both the milk yield and somatic cell count. However, there was no association between *IL17A*-1835 G>A SNP sites and any measured traits. In the future, large-scale and well-designed studies must be conducted because these SNPs have the potential to change gene expression. They will need to be investigated further regarding their impact on physiological and practical importance.

## Figures and Tables

**Table 1:** Ingredients and nutrient composition of the diet (dry matter basis).

Item	Percentage
Ingredient, % of DM	
Alfalfa hay	25.31
Corn silage	28.50
Oat hay	6.16
Ground corn	17.48
Soybean meal	5.26
Cottonseed meal	4.06
Distillers dried grains with solubles	5.30
Barely	5.18
Limestone	0.32
NaHCO_3_	0.36
NaCl	0.31
CaHPO_4_	0.56
Premix	1.20
Composition, % of DM	
Crude protein	15.02
Ether extract	3.96
Neutral detergent fiber	41.11
Acid detergent fiber	22.04
Calcium	0.80
Phosphorus	0.44
NEL^2^ Mcal/kg	6.29

The premix provided the following per kg of the concentrate: VA 300,000 IU, VD 385,000 IU, VE 1455 IU, nicotinic acid 550 mg, Cu 770 mg, Mn 930 mg, Fe 1200 mg, Zn 3600 mg, Se 21 mg, I 50 mg, Co 12 mg. NEL was a calculated value according to NRC (2001), whereas the others were measured values.

**Table 2:** The primer sequences, production size, and annealing temperature of PCR amplification for the *IL17A* gene.

Primer.	(5′→3′) Primer of Sequences	Size of Production (bp)	Position of Production	Annealing Temperature (°C)
P1	F: GGAGTGTGGTGGAGGGTAAAA	778	−2060~−1282	57
	R: CCTATTCCCAAACCTACTGCCA			
P2	F: AGTTGAATCACTTTGCTTTACAGT	845	−1413~−568	55
	R: ACATCTACTCTGCCTGAGGAAC			
P3	F: TCACCACCTTTCTGCAGTCTC	775	−748~27	57.5
	R: TGAACTTGTGCTCGCTGTGA			
P4	F: GGGGCGGTTTTTCTTTGACC	457	−143~314	58
	R: TGTGTGGTTTAGCCCCAGTC			
P5	F: GCCATGGTCCTAATGTCACT	505	1063–1568	56
	R: TGGCTCTTCCAGGTTTGACA			
P6	F: AGGAATTCACTTTCTTCCTGGCTT	759	2747–3506	57
	R: TGCTGTCTCTCTTGTAATGCCT			

**Table 3:** Genotypic and allelic frequency of *IL17A* gene and HWE test for each SNP.

SNP Locus	Genotype	Genotype Frequency	Sample Number	Allele	Allele Frequency	χ^2^ Value for the H-W Test	Pearson’s *p* Test
***IL17A* (-398)**	AA	0.247	239	A	0.514	4.478	0.034
	AT	0.534	516	T	0.486		
	TT	0.219	212				
***IL17A* (-1578)**	AA	0.109	89	A	0.374	13.742	0.000
	AG	0.529	431	G	0.626		
	GG	0.362	295				
***IL17A* (-1835)**	AA	0.026	21	A	0.126	6.778	0.009
	AG	0.200	162	G	0.874		
	GG	0.774	627				
***IL17A (*3164)**	CC	0.398	364	C	0.637	1.139	0.286
	CT	0.479	438	T	0.363		
	TT	0.123	113				
***IL17A* (3409)**	CC	0.383	363	C	0.488	316.527	0.000
	CG	0.211	200	G	0.512		
	GG	0.406	385				

HWE: Hardy–Weinberg equilibrium.

**Table 4:** Estimated haplotype frequency of 5 mutations in *IL17A* gene.

Haplotype	*IL17A* *-1578A>G*	*IL17A* *-1835G>A*	*IL17A* *-398T>A*	*IL17A* *3164T>C*	*IL17A* *3409G>C*	Sample	Estimated Frequency
1	G	G	A	C	C	384	0.303
2	A	G	T	T	G	356	0.281
3	G	G	A	C	G	111	0.087
4	G	G	T	T	G	80	0.063
5	G	G	T	C	C	78	0.062
6	A	G	A	C	C	61	0.048
7	G	A	A	C	C	48	0.038
8	A	A	T	T	G	24	0.019
9	G	A	T	C	C	24	0.019
10	A	G	T	C	C	18	0.015
11	G	A	A	C	G	16	0.013
12	G	A	T	C	G	16	0.012
13	A	A	A	C	C	16	0.012
14	G	G	T	C	G	14	0.011
15	A	G	A	C	G	10	0.008
16	A	A	T	C	C	4	0.003
17	A	A	A	C	G	4	0.003
18	A	A	T	C	G	2	0.002
19	G	A	T	T	G	1	0.001
Total						1267	1

**Table 5:** Effects of different genotypes based on *IL17A* gene with lactating performance and SCS in Holstein.

SNP Locus	Genotypes	Record Number	Tested Day Milk Yield (kg)	Milk Fat Percentage (%)	Milk Protein Percentage (%)	Somatic Cell Score
***IL17A-1578A>G***	**AA**	827	34.35 ± 0.37 ^b^	3.57 ± 0.03	3.21 ± 0.01	2.91 ± 0.08 ^a^
	**GA**	3984	35.03 ± 0.17 ^a^	3.65 ± 0.01	3.23 ± 0.01	2.75 ± 0.03 ^b^
	**GG**	2661	34.82 ± 0.21 ^ab^	3.67 ± 0.02	3.26 ± 0.01	2.69 ± 0.04 ^b^
	**Total**	7472	34.97 ± 0.11	3.64 ± 0.01	3.24 ± 0.00	2.76 ± 0.02
	***F* value**		5.583 **	1.622	0.385	3.486 *
***IL17A-1835G>A***	**AA**	209	36.14 ± 0.75	3.71 ± 0.06	3.21 ± 0.02	3.19 ± 0.15
	**GA**	1445	35.28 ± 0.28	3.64 ± 0.02	3.21 ± 0.01	2.67 ± 0.06
	**GG**	5894	35.09 ± 0.14	3.63 ± 0.01	3.23 ± 0.00	2.80 ± 0.03
	***F* value**		1.538	1.098	1.146	1.846
***IL17A-398T>A***	**AA**	2247	35.36 ± 0.24 ^a^	3.63 ± 0.02	3.25 ± 0.01	2.71 ± 0.04
	**TA**	4859	35.01 ± 0.15 ^ab^	3.63 ± 0.01	3.22 ± 0.01	2.80 ± 0.03
	**TT**	1813	34.62 ± 0.25 ^b^	3.69 ± 0.02	3.23 ± 0.01	2.73 ± 0.05
	***F* value**		5.692 **	1.988	0.105	1.188
***IL17A 3164T>C***	**CC**	3487	35.40 ± 0.19 ^a^	3.62 ± 0.02	3.24 ± 0.01	2.71 ± 0.04
	**TC**	4041	35.14 ± 0.17 ^a^	3.63 ± 0.01	3.21 ± 0.01	2.84 ± 0.03
	**TT**	986	34.37 ± 0.34 ^b^	3.67 ± 0.03	3.24 ± 0.01	2.74 ± 0.06
	***F* value**		3.948 **	0.592	1.129	0.199
***IL17A 3409G>C***	**CC**	3480	35.38 ± 0.19 ^a^	3.63 ± 0.02	3.24 ± 0.01	2.70 ± 0.04
	**GC**	1972	35.08 ± 0.24 ^ab^	3.63 ± 0.02	3.22 ± 0.01	2.69 ± 0.05
	**GG**	3267	34.82 ± 0.19 ^b^	3.65 ± 0.02	3.22 ± 0.01	2.88 ± 0.04
	***F* value**		4.711 **	0.46	0.195	0.262

The *F* test is used in the analysis of variance. The specific values were calculated using the *F*-test formula. TDMY is test-day milk yield; SCS is somatic cell score; **: *p* < 0.01; a,b,c,d differences in the same column are significant at *p* < 0.05.

## Data Availability

Not applicable.
